# Predicting Behavior With Implicit Measures: Disillusioning Findings, Reasonable Explanations, and Sophisticated Solutions

**DOI:** 10.3389/fpsyg.2019.02483

**Published:** 2019-11-08

**Authors:** Franziska Meissner, Laura Anne Grigutsch, Nicolas Koranyi, Florian Müller, Klaus Rothermund

**Affiliations:** ^1^General Psychology II, Institute of Psychology, Friedrich Schiller University Jena, Jena, Germany; ^2^Department for the Psychology of Human Movement and Sport, Institute for Sports Science, Friedrich Schiller University Jena, Jena, Germany

**Keywords:** implicit measures, predictive validity, IAT, attitude-behavior gap, multinomial processing tree models, wanting vs. liking, propositions vs. associations, context-dependency

## Abstract

Two decades ago, the introduction of the Implicit Association Test (IAT) sparked enthusiastic reactions. With implicit measures like the IAT, researchers hoped to finally be able to bridge the gap between self-reported attitudes on one hand and behavior on the other. Twenty years of research and several meta-analyses later, however, we have to conclude that neither the IAT nor its derivatives have fulfilled these expectations. Their predictive value for behavioral criteria is weak and their incremental validity over and above self-report measures is negligible. In our review, we present an overview of explanations for these unsatisfactory findings and delineate promising ways forward. Over the years, several reasons for the IAT’s weak predictive validity have been proposed. They point to four potentially problematic features: First, the IAT is by no means a pure measure of individual differences in associations but suffers from extraneous influences like recoding. Hence, the predictive validity of IAT-scores should not be confused with the predictive validity of associations. Second, with the IAT, we usually aim to measure evaluation (“liking”) instead of motivation (“wanting”). Yet, behavior might be determined much more often by the latter than the former. Third, the IAT focuses on measuring associations instead of propositional beliefs and thus taps into a construct that might be too unspecific to account for behavior. Finally, studies on predictive validity are often characterized by a mismatch between predictor and criterion (e.g., while behavior is highly context-specific, the IAT usually takes into account neither the situation nor the domain). Recent research, however, also revealed advances addressing each of these problems, namely (1) procedural and analytical advances to control for recoding in the IAT, (2) measurement procedures to assess implicit wanting, (3) measurement procedures to assess implicit beliefs, and (4) approaches to increase the fit between implicit measures and behavioral criteria (e.g., by incorporating contextual information). Implicit measures like the IAT hold an enormous potential. In order to allow them to fulfill this potential, however, we have to refine our understanding of these measures, and we should incorporate recent conceptual and methodological advancements. This review provides specific recommendations on how to do so.

Why does he act like this? Why does she not do what she intended to do? In our everyday life, we often try to find explanations for the behavior of others, and of ourselves, respectively. Explaining and predicting behavior is also of key interest across all fields of scientific psychology, especially when it comes to deviations between individuals’ actual behavior and the attitudes, goals, or values held by these very individuals. Why do people discriminate although they report to hold egalitarian values? Why do they not quit smoking although they know that smoking is bad? Why is there a gap between people’s self-reported attitudes and actual behavior?

Dual-process or dual-system models attribute seemingly inconsistent behavior to the triumph of an impulsive system over a reflective system of behavior control (e.g., Strack and Deutsch, 2004; Hofmann et al., 2009; Kahneman, 2011). The notion that the prediction of behavior could be improved considerably if one succeeds in measuring the processes of the impulsive system (Hofmann et al., 2007; Friese et al., 2008; Hofmann and Friese, 2008) fueled research applying so-called implicit measures of attitudes. The most popular of these measures, the Implicit Association Test (IAT, Greenwald et al., 1998) evoked enthusiastic hopes regarding its predictive value. Unfortunately, however, the IAT and its derivatives have not met these expectations.

In this article, we review findings illustrating reasons for the IAT’s unsatisfying predictive value, as well as promising ways forward. We will outline that in order to improve the predictive power of implicit measures, differentiation is key. We will argue that future research should put more emphasis on the underlying processes and concepts behind these measures. We begin with sketching the discrepancy between individuals’ behaviors and their self-expressed attitudes. We then summarize the (mostly unsatisfying) attempts to close this attitude-behavior gap with the help of implicit measures. In the main part of this article, we identify features of implicit measures that are responsible for their weak predictive validity. We review findings illustrating each of these problematic aspects along with specific, sophisticated solutions providing promising directions for future research.

## The Attitude-Behavior Gap and Implicit Measures

Attitudes and values that people express are often in conflict with their actual behavior. Indeed, although widely postulated to be associated with cognitive processes, judgments, and most importantly, behavior (e.g., Katz, 1960; Fazio et al., 1983; Ajzen, 1991), self-reported attitudes show weak predictive validity at best (with correlation coefficients being “rarely” above *r* = 0.30, Wicker, 1969; see also Kraus, 1995, who found an average *r* = 0.38). How can we close this *attitude-behavior gap*? A prominent way forward relied on the assumption that people might not be able to report on their mental processes in an accurate fashion (e.g., Nisbett and Wilson, 1977), implying that self-reports can never achieve convincing predictive value. Instead, “introspectively unidentified (or inaccurately identified) traces of past experience” (Greenwald and Banaji, 1995, p. 5) were proposed to be more crucial precursors of behavior. In this regard, dual process or dual system models posit that parts of human behavior can only be explained with processes that operate below the threshold of personal control and awareness (e.g., Strack and Deutsch, 2004; Hofmann et al., 2009; Kahneman, 2011), a view that fueled the interest in the “sub”-personal level of behavior control.

Over the last decades, a number of new attitude measurement procedures were introduced that aimed to tap into these processes since they do not rely on introspection (e.g., the IAT, Greenwald et al., 1998; the Affective Priming Paradigm, Fazio et al., 1986; the Affect Misattribution Procedure, Payne et al., 2005; for overviews, see Teige-Mocigemba et al., 2010; Wentura and Degner, 2010; Gawronski and De Houwer, 2014; Gawronski and Hahn, 2019). Although differing in their procedural details, all of these measurement procedures involve computerized tasks requiring individuals to quickly execute a specific response to a set of stimuli. The performance in these tasks is then influenced by stimulus-response compatibility due to the automatic evaluations of these stimuli (De Houwer, 2001, 2003a). Hence, the scores obtained from the observed performance are interpreted in terms of attitude strength. Compared to self-report measures, these measurement procedures were assumed to provide little opportunity to control responding, preventing an influence of deliberate manipulation attempts and self-presentational concerns (e.g., Fazio et al., 1986; Greenwald et al., 1998). Some even argued that these procedures succeed in measuring a unique construct (*implicit attitude*) that is introspectively less accessible and thus distinct from the construct captured in self-report measures (*explicit attitude*; Greenwald and Banaji, 1995; Wilson et al., 2000; but see Fazio, 2007, for a different view). Accordingly, researchers often use *implicit measures* and *explicit measures* as labels for these measurement procedures. Not surprisingly, implicit measures, first and foremost the IAT (Greenwald et al., 1998), were embraced by the scientific community since they came along with the potential to measure the hidden forces of behavior. The hope was that they would finally enable researchers to understand and to predict individual behavior over and above self-report measures.

Unfortunately, the predictive validity of the IAT fell short of these expectations. Meta-analytic findings (Greenwald et al., 2009; Oswald et al., 2013; Kurdi et al., 2019) suggest that the implicit-criterion correlation (ICC) is unsatisfactorily low (average *r*_ICC_ = 0.27, Greenwald et al., 2009; average *r*_ICC_ = 0.14, Oswald et al., 2013; 90-percent prediction interval for ICCs from *r* = −0.14 to *r* = 0.32; Kurdi et al., 2019). Equally upsetting is the fact that the incremental predictive validity over and above self-report measures is obviously negligible (i.e., ranging between 1 and 5%; Greenwald et al., 2009; Oswald et al., 2013; Kurdi et al., 2019). Such a disappointingly low predictive validity is a frustrating state of affairs, especially because it was the low predictive value of self-reported attitudes that initiated the development of implicit measures like the IAT in the first place.

What are the reasons for the weak relationship between implicit measures and behavioral criteria? An obvious candidate is a potential lack of internal consistency in the predictor variables. Unfortunately, reporting reliability coefficients is by no means the rule for studies on predictive validity. Nevertheless, over time, the picture emerged that implicit measures often suffer from low internal consistency (for overviews, see Gawronski and De Houwer, 2014; Gawronski and Hahn, 2019). High amounts of measurement error in the resulting scores, however, shuffle the rank order of individuals, and thus constitute a serious problem when it comes to predicting relevant criteria like behavior (for an elaboration on further consequences of low reliability, see LeBel and Paunonen, 2011; but see also De Schryver et al., 2016). Reliability, however, seems to be less of an issue for the most popular implicit measure, the IAT (Greenwald et al., 1998). On the contrary, IAT scores typically achieve acceptable levels of reliability, and outperform other implicit measures in terms of internal consistency and test-retest reliability (e.g., Nosek et al., 2007; Gawronski and De Houwer, 2014; Gawronski and Hahn, 2019). Note, however, that it has also been suggested that the comparatively high internal consistency of the IAT might be due to systematic error variance (so-called *method variance*; see below) rather than construct-related variance (e.g., Teige-Mocigemba et al., 2010; Kraus and Scholderer, 2015). If this holds true, given that method-related variance is unlikely to explain behavior, it is not surprising that the IAT’s predictive validity turned out to be bounded. So, even for the IAT, (a lack of) reliability might be part of the problem.

For the remainder of this article, however, we put reliability issues aside, and instead focus on four potentially problematic features of implicit measures and, in particular, of the IAT. We will review relevant findings as well as theoretical considerations, and we will outline that each of these features might be responsible for the IAT’s weak predictive validity: First, the IAT turned out not to be a process-pure measure of attitudes. Instead, non-attitudinal influences also play a role (for an overview of these and other shortcomings of the IAT and its derivatives, see Fiedler et al., 2006; Teige-Mocigemba et al., 2010; Gawronski and Hahn, 2019). If we want to predict individual’s behavior, we have to filter out this construct-irrelevant variance. Second, the IAT (just as most implicit measures) focuses on evaluation rather than motivation. However, people do not always want what they like (and vice versa). We should thus not confuse liking with wanting (e.g., Tibboel et al., 2015b), and in many situations, the latter might actually be more relevant in driving behavior than the former. Third, as disclosed by its very name, the IAT was introduced to quantify associations. Associations, however, might be too unspecific to unambiguously relate to and account for a particular behavior in a specific situation. Instead, (implicit) propositional beliefs could be a more plausible precursor of behavior (e.g., Hughes et al., 2011). Finally, when applying the IAT researchers typically aim at assessing attitudes or stereotypes globally, that is, in a context-independent fashion. Mental representations of attitudes and stereotypes, however, are highly context-dependent. Similarly, real-life behavior does not occur in a situational vacuum. The predictive validity of implicit measures like the IAT might thus be improved by increasing the match between predictor and criterion (i.e., overcoming the lack of specificity in the predictor by incorporating contextual information).

Note that we do not want to imply any order or priority with regard to these four issues. We will outline that each of them could be responsible for the IAT’s weak predictive power. This however does not exclude the possibility that researchers might have to address several (if not all) of these features in order to achieve the desired results. In the remainder of this article, we explain all of these potentially problematic features in detail, along with promising ways forward and specific recommendations for future research.

## Issue 1: Extraneous Influences on Implicit Measures

Implicit measures (just like explicit ones) should not be understood as process-pure measures of attitudes. They are characterized by additional, non-attitudinal influences, and this kind of error variance reduces their predictive validity. This also applies to the IAT (Greenwald et al., 1998), one of the most popular implicit measures.

The IAT involves two binary classification tasks, a target task and an attribute task, that have to be performed with two response keys. Importantly, the key assignment varies across the two IAT test blocks. In the *compatible* block, participants are instructed to press one key for the positively evaluated target category (e.g., flower) as well as the positive pole of the attribute dimension (e.g., positive), and to press the other key for the more negatively evaluated target category (e.g., insect) as well as the negative pole of the attribute dimension (e.g., negative). In the *incompatible block*, negative targets and positive attributes are assigned to the same key (and positive targets and negative attributes to the other key, respectively). Participants typically respond faster and more accurate in compatible compared to incompatible IAT blocks. The performance difference between compatible and incompatible blocks (*compatibility effect*, *IAT effect,* or *IAT score*) is then interpreted as a measure for the strength of associations between the respective categories (Greenwald et al., 1998)^1^.

During the 20 years since its introduction, however, numerous findings challenged the IAT’s construct validity (for an overview, see Teige-Mocigemba et al., 2010). An illustrative example is the finding that content-unrelated IATs (i.e., two IATs that involve non-overlapping target concepts) share a considerable amount of variance (so-called *method variance*; e.g., Greenwald et al., 1998; McFarland and Crouch, 2002; Mierke and Klauer, 2003; Back et al., 2005; Klauer et al., 2010). In search for an explanation for this shared method variance, several groups of researchers proposed attitude-unrelated processes that affect IAT responding, such as general processing speed (McFarland and Crouch, 2002; Blanton et al., 2006) or executive functions like task-switching ability (Klauer et al., 2010; Ito et al., 2015). Another potential flaw of the IAT is the fact that it suffers from usually unwanted block order effects: IAT scores turn out to be larger if participants started with the compatible block (e.g., Greenwald et al., 1998; Nosek et al., 2005; for a possible explanation, see Klauer and Mierke, 2005). Finally, IAT scores do not only reflect the valence of the target categories but can also be influenced by stimulus effects (e.g., Steffens and Plewe, 2001; Mitchell et al., 2003; Govan and Williams, 2004; Bluemke and Friese, 2006; Gast and Rothermund, 2010).

Summing up, numerous studies revealed that IAT scores do not reflect pure attitude strength but also contain systematic error variance. The mere amount and variety of different findings (for an overview, see Teige-Mocigemba et al., 2010) is not particularly easy to grasp. In the following, however, we outline that there is a common core behind these additional processes: *recoding* (e.g., De Houwer, 2003b; Wentura and Rothermund, 2007; Rothermund et al., 2009).

### The Role of Recoding in the Implicit Association Test

Although instructed to perform a double categorization task, participants can often easily simplify the IAT through so-called task *recoding*. Recoding refers to a combination of targets and attributes to superordinate categories. It is based on some degree of similarity in the IAT’s stimulus material, that is, some feature that targets and attributes share. In a flower-insect IAT, for example, participants can profoundly simplify the task in the compatible block by categorizing each stimulus according to its valence, and ignoring the fact that some stimuli should actually be categorized according to their identity (i.e., flowers vs. insects). If the task is recoded in this sense, the compatible block involves only one and the same binary decision (i.e., is the current stimulus positive or negative?). In the incompatible block, on the other hand, the incongruent response assignment prevents recoding. Here, participants have no choice but to follow the instructed, rather difficult double categorization task (i.e., flowers vs. insects, and positive vs. negative).

Recoding thus results in a substantial block difference in task difficulty, and therefore accounts for the observed block difference in response times and error rates (e.g., Rothermund et al., 2009). Remarkably, it has been shown that even in the absence of any category-based associations, recoding processes produce significant IAT scores (e.g., Mierke and Klauer, 2003; Rothermund and Wentura, 2004; De Houwer et al., 2005).

Note that recoding must not be based on stimulus valence. Instead, every feature that is shared by targets and attributes might be used to form superordinate categories (e.g., salience, familiarity, valence, or even perceptual features like color or shape; Rothermund et al., 2009; see also Mierke and Klauer, 2003; Rothermund and Wentura, 2004; De Houwer et al., 2005; Kinoshita and Peek-O’Leary, 2006; Chang and Mitchell, 2009)^2^. Whether it is valence, salience or some other feature, if the task was recoded, responses are based on the shared feature, and thus necessarily unrelated to the (attitudes toward the) nominal categories (e.g., faces in a Black-White IAT are no longer processed as Black vs. White but rather as more vs. less salient, Kinoshita and Peek-O’Leary, 2005). Even more important, recoding should not be understood as a more or less constant error that boosts IAT scores equally for everyone. Instead, there might be inter-individual differences in recoding [e.g., due to individual differences in familiarity, Greenwald et al., 1998 (Exp. 2), salience, Rothermund and Wentura, 2004 (Exp.’s 2A and 2B), or fluid intelligence, von Stülpnagel and Steffens, 2010] that can be unrelated to the to-be-measured attitudes. In this sense, recoding represents a source of variance that might distort the predictive validity of the IAT score for behavioral criteria. For more detailed elaborations on this issue, and for findings of recoding being unrelated to the construct of interest (i.e., attitudes), we refer to the work of Meissner and Rothermund (2013, 2015a,b).

Recoding can be understood as the most crucial extraneous influence in the IAT because it can account for other extraneous influences that were identified throughout the last couple of years. As an example, consider the negative correlation of IAT scores with task-switching ability (e.g., Klauer et al., 2010). Task-switching ability, that is, high cognitive flexibility, enables fast and effortless switches between two tasks. Therefore, high switching ability reduces switch costs between the two classification tasks in the IAT (i.e., between target and attribute classification). This is especially helpful in the incompatible block of the IAT, where participants have to perform the double categorization task. In the compatible block, on the other hand, the task can be simplified by recoding. If they engage in recoding, people no longer switch between the two tasks: By combining pairs of targets and attributes into superordinate categories, they now perform only a single binary decision. Consequently, people with high vs. low switching ability will perform equally well in the compatible IAT block. Recoding thus results in a negative correlation of switching ability and IAT scores. Similarly, the relationship between IAT scores and general processing speed (e.g., McFarland and Crouch, 2002) can be explained with recoding as well. Finally, it has been shown that task recoding can also account for stimulus effects in the IAT (e.g., Gast and Rothermund, 2010).

At this point, it should be clear that the IAT score should be understood as a mixture of both relevant influences (e.g., associations) and irrelevant influences, first and foremost, recoding. If researchers want to increase the IAT’s predictive validity, they should thus try to separate effects of associations from the influence of recoding. In the past few years, two different approaches were introduced that claim to do so: The first approach aims at minimizing recoding processes by modifying the IAT procedure. The second approach disentangles associations and recoding processes with the help of multinomial modeling. In the following, we will present a short overview of these suggestions.

### A Solution: Dropping the Block Structure

As outlined above, recoding effects in the IAT can be traced back to its characteristic structure: the arrangement of trials in (compatible vs. incompatible) blocks. When it comes to reducing the influence of recoding, an obvious possible solution is thus to simply omit this structure. In this regard, several variants of the IAT have been introduced that dropped the characteristic block structure, and varied response compatibility within one test block instead: the Single-Block IAT (SB-IAT, Teige-Mocigemba et al., 2008) and the Recoding-Free IAT (IAT-RF, Rothermund et al., 2009)^3^. While the category-response assignment is constant throughout a block of trials in the standard IAT, it varies randomly from trial to trial in the newer IAT variants. Consequently, scores in those procedures are obtained by computing performance differences between compatible and incompatible *trials* rather than between compatible and incompatible *blocks*.

In these IAT variants, participants are informed about the current category-response assignment either by simply showing it shortly before the stimulus appears (IAT-RF) or by using stimulus position as a cue (with an appearance in the upper half of the screen signaling a compatible assignment, and an appearance in the lower half of the screen indicating an incompatible assignment; SB-IAT). Crucially, the upcoming category-response assignment is not predictable. Consequently, a stable and efficient recoding strategy specifically for the compatible assignment becomes much harder than in the standard IAT. This reasoning was supported by Rothermund et al. (2009) who found that dropping the IAT’s block structure successfully reduces switch cost asymmetries, a marker of recoding processes.

Besides reducing the effects of recoding, the block-free IAT variants come with some further advantages. For example, block order effects which usually influence conclusions in the standard IAT (e.g., Greenwald et al., 1998) are no longer an issue. Furthermore, the newer IAT variants eliminate method-related variance (Teige-Mocigemba et al., 2008) and stimulus effects (Gast and Rothermund, 2010). These findings also support the assumption that recoding is one of the most crucial validity threats of the IAT. Finally, the block-free IAT variants are not only correlated with behavioral criteria (Teige-Mocigemba et al., 2008; Houben et al., 2009), there is also evidence that dropping the block structure of the IAT can actually improve its predictive validity (Kraus and Scholderer, 2015).

Despite these strengths of SB-IAT and IAT-RF, the strategy to minimize recoding effects by dropping the IAT’s block structure bears the risk to miss potentially interesting effects. Although recoding processes do not represent the construct that researchers typically attempt to measure when employing the IAT, they might nevertheless represent variance that is related to criteria of interest. It has been proposed, for example, that recoding could reflect explicit attitudes (Rothermund et al., 2009) and that occasionally, it might be related to relevant criteria (e.g., behavior; Rothermund et al., 2005; Teige-Mocigemba et al., 2008).

The second approach also dealing with the problem of recoding follows a different rationale. Instead of trying to reduce the influence of recoding, it assumes that IAT scores result from a mixture of different processes. As summarized in the following section, this approach then relies on mathematical modeling to measure each of these processes. This allows researchers to separately examine the predictive power of both construct-related and method-related variance due to recoding.

### Another Solution: Adopting a Modeling Approach

Recently, a multinomial processing tree model has been introduced that enables a remarkably fine-grained analysis of the IAT: The ReAL model (Meissner and Rothermund, 2013). Most importantly, this model successfully disentangles the effects of evaluative associations from the distorting influence of task recoding. In this section, we provide a brief overview of the ReAL model’s basic idea, and we review relevant findings concerning (improvements on) the IAT’s validity.

The ReAL model assumes that the observable responses in the IAT (i.e., correct and incorrect responses in compatible and incompatible blocks) result from the interplay of specific unobservable processes (e.g., associations and recoding; see below). These processes are represented by separate model parameters; their assumed interplay is displayed in a tree architecture (i.e., the *multinomial processing tree*). Based on observed response patterns, algorithms estimate values for the model parameters which are then interpreted as measures for the respective cognitive processes (for mathematical details on multinomial processing tree models, see Riefer and Batchelder, 1988; Hu and Batchelder, 1994; Batchelder and Riefer, 1999; for reviews of applications, see Erdfelder et al., 2009; Klauer et al., 2012).

The ReAL model distinguishes three different processes: recoding (*Re*), evaluative associations (*A*) and the resource-consuming label-based identification of the correct response (*L*). The tree structure incorporates theoretical assumptions concerning these processes. For example, the ReAL model assumes that task recoding determines responding for both targets and attributes but only in one of the IAT blocks (i.e., in the compatible block)^4^. Evaluative associations, on the other hand, are assumed to influence responding in both compatible and incompatible blocks but they should be triggered only in target trials, not in attribute trials (reflecting the understanding of attitudes as evaluative associations triggered by an attitude object, not vice versa; Fazio et al., 1986; see also Anderson, 1983).

As a multinomial model, the ReAL model is able to disentangle multiple cognitive processes accounting for the same observable response (Batchelder and Riefer, 1999). First and foremost, the ReAL model controls for the effects of recoding by measuring them in a separate model parameter (which clearly represents a unique feature as compared to other mathematical models for the IAT; e.g., the quad model, Conrey et al., 2005; or the diffusion model, Klauer et al., 2007). Besides addressing the problem of recoding, the ReAL model comes with another advantage: While IAT scores only reflect *relative* preferences (which could be problematic; for an overview, see Teige-Mocigemba et al., 2010), the ReAL model provides separate association parameters for each of the two target categories. Consequently, the model can successfully handle situations where both attitude objects trigger equally strong positive, negative, or neutral associations. Note that the conventional IAT score would only yield a null effect in these cases (i.e., no preference).

Numerous studies revealed that the ReAL model parameters are valid measures of the processes they stand for (Meissner and Rothermund, 2013; Meissner and Rothermund, 2015a,b; see also Koranyi and Meissner, 2015; Jin, 2016). Most importantly, the ReAL model’s association parameters reflect the direction and the strength of evaluative associations for each of the two target concepts (Meissner and Rothermund, 2013). This holds true even in IAT applications where recoding processes pushed the overall IAT score in the opposite direction (Meissner and Rothermund, 2015a). The association parameters turned out to be sensitive to manipulations of evaluation (Meissner and Rothermund, 2013) but immune against artificial, non-evaluative influences (i.e., salience asymmetries and modality match effects; Meissner and Rothermund, 2015a,b). Additionally, and in line with theoretical considerations (e.g., Fazio and Towles-Schwen, 1999), association parameters correlated with self-reported attitudes in non-sensitive attitude domains (consumer preferences; Meissner and Rothermund, 2013). Finally, Meissner and Rothermund (2013) also tested the predictive validity of the model’s association parameters. As expected, the amount of chocolate consumed while watching a video was successfully predicted by the ReAL model’s association parameter (estimated from the response pattern in a fruit-chocolate IAT). Note that the behavior was unrelated to the recoding parameter and also unrelated to the conventional IAT score (i.e., the D score; Meissner and Rothermund, 2013). When it comes to increasing the IAT’s predictive validity, an application of the ReAL model thus constitutes a promising step forward. Given the recent developments in the field of multinomial processing tree models (i.e., allowing the incorporation of response time data, Heck and Erdfelder, 2016; Klauer and Kellen, 2018; and a sophisticated treatment of possible parameter heterogeneity, e.g., Klauer, 2010; Matzke et al., 2015) further improvements are to be expected. Given that the ReAL model has already outperformed the IAT score with regard to construct validity in a number of studies (e.g., Meissner and Rothermund, 2013, 2015a,b), we recommend researchers to consider an application of the ReAL model as an alternative, or at least as an additional analysis tool for the IAT in their studies.

So, we cannot deny that extraneous influences on IAT scores like recoding do exist. However, there are promising approaches to address this problem. With procedural modifications or mathematical modeling, we can measure more validly what people actually like. But what if it is irrelevant what people like? Maybe it is more important what people *want*?

## Issue 2: Distinguishing Between Liking and Wanting

Insights from recent neuropsychological research raise the question whether evaluations are indeed the driving force behind behavioral impulses. According to the incentive salience hypothesis (Robinson and Berridge, 1993, 2001; Berridge and Robinson, 2003; Berridge, 2009), *liking* an object and *wanting* it are separable processes that are mediated by different brain substrates and are differentially affected by various factors. Whereas “liking” refers to the hedonic aspects of a stimulus (i.e., the pleasure or positive affect it causes), “wanting” is the result of the attribution of incentive salience. The latter describes a particular quality that, when added to the mental representation of a given stimulus, transforms the mere sensory percept of this stimulus to become attention-grabbing, attractive, and potent to elicit behavioral impulses of approach or consumption, which is the very essence of behavioral motivation (Berridge and Robinson, 2003; Berridge, 2009).

Importantly, while “wanting” and “liking” should generally covary (i.e., the strength of “wanting” experienced for a specific object should be proportional to the hedonic “liking” it produces), there are specific classes of stimuli and situations where the two processes can become uncoupled. The most prominent example for such a dissociation is the case of addiction, where “wanting” for the addictive drug is extremely enhanced long after it ceases to evoke hedonic experiences (i.e., “liking”), and even despite the addict’s recognition of its harmful effects (Robinson and Berridge, 1993; Stacy and Wiers, 2010). Even though momentary dissociations of “wanting” and “liking” are at the heart of many chronic clinical psychological conditions (e.g., Rømer Thomsen et al., 2015; Olney et al., 2018), they are not in themselves pathological (Dill and Holton, 2014). Rather, the closeness of the relationship between “wanting” and “liking” fluctuates in healthy individuals (Epstein et al., 2003; Hobbs et al., 2005; Dai et al., 2010, 2014; Litt et al., 2010). An illustrative example is the moment after finishing a delicious meal. While “liking” for the food will be unaltered, being satiated will reduce “wanting” more of it (Kraus and Piqueras-Fiszman, 2016; Stevenson et al., 2017). However, not only states of satiation and deprivation have differential effects on “wanting” and “liking.” It has also been shown, for instance, that stress increases “wanting” but not ‘liking’ for sweet rewards (Pool et al., 2015).

To sum up, “wanting” and “liking,” though typically highly correlated, can diverge. Whenever they do, “wanting” is much more likely to guide behavior than “liking” (Berridge et al., 1989; Peciña et al., 2003). Researchers interested in predicting behavior are therefore well advised to incorporate measures of “wanting” (Lades, 2012).

### Initial Attempts in Assessing “Wanting”

How do we measure “wanting”? Self-reports are not an advisable option. Obviously, they involve the risk of potential distortions due to self-presentational concerns, especially when it comes to sensitive topics. Apart from that, however, disentangling “wanting” and “liking” on a semantic level is complicated. Participants might fail to grasp the distinction or simply confuse the two processes since the consideration of wanting as independent from liking violates laymen’s intuition. Furthermore, as pointed out by Pool et al. (2016), it is likely that self-reported “wanting” primarily reflects expected pleasantness, and is inferred from past hedonic experiences (i.e., “liking”). Actual implicit “wanting,” on the other hand, should in principle be independent from any hedonic aspects of reward (Robinson and Berridge, 2013).

Several researchers have therefore turned to established implicit measurement procedures, most often the IAT, in order to develop a measure of implicit “wanting” as distinct from implicit “liking” (for an overview, see Tibboel et al., 2015b). By now, several IAT variants have been introduced that aim to measure implicit “wanting” for a given target dimension of interest (e.g., alcohol vs. no alcohol, smoking vs. no smoking, attractive vs. unattractive persons). All of these approaches share one basic idea. That is, in order to transform the IAT into a measure of implicit “wanting” the category labels of the evaluative attribute dimension have to be replaced with concepts representing some aspect of “wanting.” Based on the notion that “wanting” entails the urge to approach the object in question, Palfai and Ostafin (2003) for instance, introduced an IAT that employs the attribute categories “approach” and “avoidance,” with semantically related words (e.g., advance, withdraw) as stimulus material (see Kraus and Scholderer, 2015, for a similar approach using the IAT-RF). In a similar vein, Wiers et al. (2002) developed an IAT employing the attribute categories “active” and “passive” represented by arousal and sedation-related words. Tibboel et al. (2011, 2015a), on the other hand, used “I want” vs. “I do not want” as attribute categories in their IAT with positive vs. negative (e.g., holiday, pain; Tibboel et al., 2011), or motivational words (e.g., gain vs. deprivation; Tibboel et al., 2015a) as stimulus material.

However, there are reasons to doubt the validity of these attempts at creating a measure of implicit “wanting.” For example, in situations that should actually reveal a dissociation of “wanting” and “liking,” these IAT variants designed to measure “wanting” typically reveal a high overlap with “liking” measures (for an overview, see Tibboel et al., 2015b). Obviously, changing the attribute categorization task on a merely semantic level by simply replacing the category labels cannot transform the IAT into an implicit measure of “wanting.” If anything, these IATs most likely reflect semantic associations, or a “cognitive form of wanting” (Tibboel et al., 2015b, p. 189). Recently, however, a new Wanting-IAT was introduced (Koranyi et al., 2017) that can be considered a more promising way forward in multiple respects.

### A Solution: The Wanting Implicit Association Test

The basic idea of the Wanting-IAT (W-IAT, Koranyi et al., 2017) consists in endowing the attribute discrimination task with motivational character. More precisely, execution of one of the attribute responses should come to equal execution of a “wanting”-triggered consummatory response. Relative “wanting” for a pair of target concepts could then be assessed in the form of stimulus–response-compatibility effects (De Houwer, 2001, 2003a) by comparing the speed and accuracy of responses when either of the two target categories is mapped onto the established “wanting” response key.

To achieve this, several adjustments to the conventional IAT procedure are necessary. First, instead of valence (as in traditional IATs), or purely semantic meaning (as in previous attempts at creating a “wanting” IAT), the relevant criterion for the categorization of attribute stimuli in the W-IAT must consist in participants’ “wanting” for them, or lack thereof, respectively. This entails the need for a set of attribute stimuli that is potent to trigger acute bursts of “wanting,” and another that is not. Second, execution of the required response for wanted stimuli must acquire the quality of an actual “wanting”-triggered consummatory response.

As for the first requirement, it must be considered that being “wanted” is not an inherent property of any specific stimulus, but instead hinges on its interaction with the individual’s current psychological or physiological state (Zhang et al., 2009; Robinson and Berridge, 2013). Thus, to ensure “wanting” for one set of attribute stimuli in the W-IAT, a physiological need state is induced in participants before completion of the W-IAT, and one set of attribute stimuli is selected to be highly relevant for satisfying this very need. Specifically, before starting the W-IAT, participants are made thirsty with salty snacks. Attribute stimuli in the following W-IAT then consist of images of drinks (need-relevant) and neutral objects (need-irrelevant). The attribute task in the W-IAT is then to sort these stimuli into the categories “I want” (for drinks) and “I don’t want” (for neutral objects). Executing this categorization is then transformed into a consummatory response by making “I want”-key presses instrumental for need satisfaction. More precisely, whenever participants correctly press the “I want”-key in response to pictures of drinks, they gain a small amount of water for later consumption. To further increase the consummatory character of the “I want” response, this gain is signaled by immediate visual and auditory action effects: a small glass appears in the lower part of the screen, and a drinking-related sound (e.g., cork popping and/or gurgling water) is presented *via* headphones.

The potential of this new W-IAT was illustrated in a study on attraction in a mating context (Koranyi et al., 2017). Heterosexual male participants completed the previously described W-IAT procedure as well as a standard valence IAT (i.e., positive vs. negative attribute dimension). Target stimuli in both IATs were very attractive vs. less attractive faces. IAT scores should therefore reflect participants’ implicit “wanting” and “liking” for those faces. Importantly, however, half of the target faces were male, while the other half was female. The study revealed the expected dissociation of “wanting” and “liking”: Both attractive male and attractive female stimuli elicited “liking” (as measured by the standard valence IAT) but only attractive female (not male) faces triggered “wanting” (as measured by the W-IAT). In other words, the results show a general positive evaluation of attractiveness, irrespective of gender, while an implicit wanting can only be found for attractive opposite-sex targets (Dai et al., 2010).

Note that this study additionally employed another version of the wanting IAT, namely a variant that used only the semantic labels “I want” and “I do not want” without bestowing any additional motivational meaning onto the attribute discrimination task. This variant yielded the same effects as the standard valence IAT. This detail in the results underpins the assumption that purely semantic “wanting” measures fail to dissociate themselves from comparable measures of “liking” (c.f., Tibboel et al., 2011, 2015a). The findings of Koranyi et al. (2017) thus suggest that an implicit measure of “wanting” should establish the motivational quality of relevant responses.

The validity of the W-IAT was further corroborated in a study that compared smokers’ and nonsmokers’ “wanting” and “liking” for smoking cues (Grigutsch et al., 2019). This study revealed that the W-IAT is better suited to discriminate between smokers and nonsmokers than a standard valence IAT tapping “liking.” Specifically, W-IAT scores were positive for smokers but negative for nonsmokers, while “liking”-IAT scores were negative for both groups. Furthermore, in line with the notion of an addiction-related decoupling of “wanting” and “liking,” the correlation of W-IAT and “liking”-IAT was significantly weaker for smokers than for nonsmokers. In contrast to previous attempts at this matter, the W-IAT thus proved to measure actual “wanting” instead of purely semantic associations (c.f., Palfai and Ostafin, 2003; Tibboel et al., 2011, 2015a) both in situations where “liking” is high (Koranyi et al., 2017) and in situations where “liking” is low (Grigutsch et al., 2019).

So, when behavior is not in line with attitudes or values, this might be due to a dissociation of “wanting” and “liking.” Implicit measures of “wanting,” first and foremost those that actually realize a wanting quality (i.e., the W-IAT), are a promising alternative to existing measures of implicit “liking” when it comes to closing the attitude-behavior gap.

## Issue 3: Focus on Associations Versus Beliefs

Interestingly, when researchers started to engage in the development of implicit measurement procedures, many also changed the focus with regard to the construct they attempted to measure. Self-report measures assessed complex personal beliefs that can be expressed in propositional statements. With the development of the IAT and other implicit measures (e.g., Affective Priming, Fazio et al., 1986), the concept of beliefs took a backseat in many studies. A lot of researchers now focused on measuring associations, that is, the mental connection between an object and a given attribute (e.g., positive or negative valence). Such an associative link, however, is unspecific in its nature, and admits several meanings.

### Ambiguity of Associations

From the literature on evaluative learning, we know that it is not only mere associative co-occurrence that determines valence transfer from an unconditioned stimulus (US) to a conditioned stimulus (CS). Instead, relational qualifiers moderate this relationship. For example, experiencing a neutral person (CS) together with a positively evaluated person (US) will result in positive evaluations of the CS if the relationship between the two persons is framed as friendship. If the relation between the two is described as being antagonistic, however, presenting them together will lead to a negative evaluation of the CS (Fiedler and Unkelbach, 2011; see also Peters and Gawronski, 2011; Förderer and Unkelbach, 2012; Zanon et al., 2014; Van Dessel et al., 2018).

Associations as they should be measured by implicit measurement procedures do not contain qualitative relational information. Therefore, a given association between two concepts can reflect different, sometimes even opposite beliefs. For example, “I” and “good” may be associated either because I believe that I am good, or because I believe that I am no good, or because I would desperately like to be good, or because I know that others would like me to be good (see also De Houwer, 2014; De Houwer et al., 2015). This raises the question whether the weak predictive validity of implicit measures of associations (e.g., Greenwald et al., 2009; Oswald et al., 2013) is due to the fact that associations are simply unspecific. Some researchers even argued that the attempt to predict behavior with associations must fail because all information stored in memory is inherently propositional (e.g., Hughes et al., 2011; De Houwer, 2014). The latter, however, is part of an ongoing debate in the literature, and we will not address it in detail in this overview. Still, what remains is that (measures of) associations are ambiguous with regard to the qualitative relation between the concepts involved, and that this could be responsible for the weak predictive validity of implicit measures. The attitude-behavior gap might be addressed more convincingly with implicit measures of propositional beliefs instead of associations.

### A Solution: Implicit Measures of Beliefs

The notion of implicit measures of beliefs represents a relatively recent development (Barnes-Holmes et al., 2010; De Houwer et al., 2015; Müller and Rothermund, 2019). Although the procedural details of these different measures vary, they all capitalize on the finding that during an evaluative processing of propositions (e.g., “Milk is not white.”) beliefs about the truth of these propositions (i.e., “False”) are activated automatically (e.g., Wiswede et al., 2013). In contrast to established implicit measures of attitudes that do not take into account the specific semantic relationship between concepts, implicit measures of beliefs allow for the assessment of complex propositions. They naturally employ more complex stimuli than traditional attitude measures, that is, combinations of stimuli including their semantic relationship, or even whole sentences. This common basis notwithstanding, these measures utilize different approaches to assess implicit beliefs, each entailing unique advantages as well as shortcomings. In the following, we provide a brief overview.

#### Implicit Relational Assessment Procedure

In each trial of the Implicit Relational Assessment Procedure (IRAP, Barnes-Holmes et al., 2010; see also Remue et al., 2013, 2014), participants are presented with two concepts that are simultaneously displayed in the top and bottom half of the screen (e.g., “I” and “nice” or “I” and “worthless”). Additionally, the IRAP highlights the propositional relationship between the two concepts by presenting a relational qualifier (e.g., “I *am* nice.” or “I *am not* worthless.”). Participants are instructed to respond to these stimuli in a specific manner across the two blocks of the task. In a first block they are to classify these stimuli as true or false (by pressing one of two keys labeled “true” and “false”) depending on whether they are *in line* with a specific belief (e.g., the belief “I am good.”). In the second block of the task, this reference belief is reversed (i.e., stimuli in line with the belief “I am no good” would require a “true” response). Additionally, in order to prevent confounding the physical location of a response key (i.e., left vs. right) and its meaning (e.g., true vs. false) key assignment is varied on a trial by trial basis.

Attesting to the fact that beliefs drive responding in the IRAP, task performance differs between both blocks. Specifically, responding in the IRAP is faster and more accurate if the response rule is in line with personal beliefs (Barnes-Holmes et al., 2010). Additionally, these effects are sensitive to changes in the relational qualifier, such as from “I am” to “I want to be” allowing for dissociation of different kinds of beliefs (e.g., uncovering differences between actual and ideal self, Remue et al., 2013, 2014) that are impervious to traditional implicit measures like the IAT.

However, due to its block-based nature, the IRAP is limited to assessing implicit beliefs toward a single set of beliefs at a time (i.e., for a given pair of blocks with their associated reference beliefs). In addition, IRAP scores have been shown to be susceptible to faking attempts (Hughes et al., 2016) and often exhibit moderate reliability only (e.g., Remue et al., 2013, 2014; see also Gawronski and De Houwer, 2014). Finally, the IRAP is also held back by substantial dropout rates in participants (more than 20% dropout is reported among university students in Remue et al., 2013; for a discussion, see De Houwer et al., 2015) – an issue that is thought to be attributable to the trial-by-trial response key reassignment.

#### Relational Responding Task

The so-called Relational Responding Task (RRT, De Houwer et al., 2015) directly addresses the issue of dropouts in the IRAP by avoiding the trial-by-trial response key reassignment. To this end, inducer trials require participants to classify synonyms of the concepts “true” and “false” by button press as either “true” or “not true” thereby constantly reinforcing the intended key meaning (De Houwer et al., 2015). On the other hand, target trials present participants with whole sentences stating certain kinds of beliefs (e.g., regarding immigrants, De Houwer et al., 2015; or smoking, Tibboel et al., 2017). Mirroring the design of the IRAP discussed above, a block specific reference belief governs which of two responses (i.e., “true” vs. “not true”) participants should give. One block requires participants to respond “as if” they held a specific belief (e.g., as if they believed that immigrants were smarter than natives). A second block then requires participants to respond “as if” they held the opposite belief (e.g., as if they believe that natives are smarter than immigrants). Consequently, the correct response to a particular target sentence is “true” in one block but “not true” in the other block.

If implicit beliefs drive responding in the RRT, task performance should differ between the two blocks. Consequently, a relative performance increase of one RRT block over the other is assumed to indicate that the individual’s beliefs are more in line with this block’s reference belief. De Houwer et al. (2015) found that implicit beliefs of Flemish participants reflect ingroup preferences: On average, they showed better performance if they should respond as if they held pro-Flemish beliefs.

As pointed out by De Houwer et al. (2015), the RRT’s structure is similar to that of the IAT. For instance, the RRT employs two binary classification tasks sharing a set of two response keys. Furthermore, it consists of two critical blocks differing with regard to the specific response rules, and its resulting global score is based on the performance difference between these blocks. Mirroring findings for the IAT, the RRT is reliable (De Houwer et al., 2015; Tibboel et al., 2017) while being less demanding on participants as indicated by markedly reduced dropout over the IRAP (4% vs. 20%, De Houwer et al., 2015). On the other hand, by virtue of these shared structural properties, the RRT runs the risk to be subject to similar flaws as the IAT (e.g., recoding). Last, but not least, the necessity to instruct participants to react to statements in line with a block specific reference belief effectively limits the RRT to the assessment of a single belief for a given measurement session (similar to the IRAP).

#### Propositional Evaluation Paradigm

A final implicit measure of beliefs employs a completely different rationale. Whereas the previously discussed procedures resemble the basic structure of the IAT, the so-called Propositional Evaluation Paradigm (PEP, Müller and Rothermund, 2019; see also Wiswede et al., 2013) is similar in design to classic priming procedures. Each PEP trial starts with a simple sentence that is presented in a word-by-word fashion (e.g., “Milk is red.”) to participants in the center of the screen. Depending on the type of trial, this is followed by a specific response prompt. On measurement trials, the response prompt (either “true” or “false”) signals to participants which of two response keys (“true”-key or “false”-key) is to be pressed. Note that the prime sentence is completely irrelevant for participants’ decision – the task is to react to the response prompt only. In contrast, on inducer trials the response prompt “? true - false?” signals participants to indicate whether the prime sentence they just saw was orthographically correct (i.e., whether or not it contained a spelling error). As in the RRT, inducer trials thus reinforce the intended key meaning.

The irrelevance of the prime sentence for participants’ reactions in the measurement trials notwithstanding, compatibility effects between the validity of the prime sentence and the required response emerge. For example, the prime sentence “Milk is red” is (obviously) false, hence, “false” is automatically activated. This in turn facilitates responding if the response prompt requires a congruent response (i.e., “false”) but interferes with responding if it requires an incongruent response (i.e., “true”) instead. Similarly, in the case of a valid (i.e., true) prime sentence faster and more accurate responding would be expected following a “true” response prompt, compared to a “false” response prompt.

However, whereas the PEP’s ability to measure beliefs concerning objectively true or false statements has been demonstrated previously (Wiswede et al., 2013) the true potential of an implicit measure of beliefs is its ability to tap into inter-individual differences in beliefs. This is especially true for beliefs related to more sensitive domains, such as beliefs concerning different social groups. As a case in point, Müller and Rothermund (2019) employed the PEP to assess individuals’ implicit beliefs concerning racism against immigrants. Therefore the items of established self-report measures of classic and modern racism (e.g., Akrami et al., 2000) served as prime sentences in the PEP. On the sample level the PEP indicated the endorsement of tolerant and welcoming beliefs about minorities and a rejection of racist beliefs. More precisely, responding with “true” was facilitated when positive beliefs about minorities were shown as primes (e.g., “A multicultural Germany would be good.”). In contrast, responding with “false” was facilitated when negative beliefs about minorities were shown as primes (e.g., “Racist groups are no longer a threat toward immigrants.”). Going beyond characteristic patterns at the sample level, the PEP proved to be sensitive to inter-individual differences in these beliefs. Specifically, more endorsement of racist attitudes on the PEP predicted (1) explicit endorsement of these statements, (2) political orientation, and (3) behavioral efforts aimed at raising money for refugees (see Müller and Rothermund, 2019, for similar findings concerning hiring discrimination and endorsement of gender stereotypes).

To summarize, processing and evaluation of complex propositional content can occur in a rapid and automatic (i.e., implicit) fashion. Recently, a number of promising implicit measures of beliefs have been introduced. Their strength lies in their ability to measure complex, propositional relationships among different concepts. This allows for more fine-grained insights as compared to measures of simple associations that have become a hallmark of established implicit measures. In our efforts at bridging the attitude-behavior gap, we should thus not rely solely on associations. We should get beliefs back on board.

## Issue 4: Lack of Fit Between Predictor and Criterion

The previous sections discussed shortcomings of the IAT and similar implicit measures and highlighted possible solutions. Note though that improving the measurement of implicit attitudes and beliefs solves only parts of the equation. It is equally important to ensure adequate measurement of the respective criterion variable.

In this section, we argue that findings of low predictive validity of implicit measures require careful consideration. If the criterion was not properly assessed, then the absence of a relation between an implicit measure and a criterion should not be understood as evidence against the measure’s validity. On the other hand, some of the reported evidence for the validity of implicit measures in predicting behavior must be discounted based on the fact that the behavior of interest was simply not assessed in the first place. Some researchers interpreted the mere presence of IAT effects as sufficient evidence for discrimination, which it is not. An IAT effect is just a response time difference in a computerized categorization task, not discriminatory behavior (e.g., Arkes and Tetlock, 2004). In our view, an effect in an implicit measure like the IAT might not even count as sufficient evidence for inferring the existence of racial biases. As the previous paragraphs have shown, these effects might be driven by various influences that can be unrelated to the categories in question (e.g., recoding that is due to salience asymmetries) or to individual attitudes (e.g., extrapersonal associations; Karpinski and Hilton, 2001). Of course, we do not want to deny that an effect in an implicit measure can provide strong evidence for inferring racial bias; however, we want to emphasize that such a claim rests on the assumption that the effect is driven by (implicit) evaluations of the categories in question. To bolster this claim, alternative explanations first have to be identified and ruled out convincingly.

In this section, however, we do not want to discuss studies that did not even assess discriminatory behaviors. Instead, we want to focus on the lack of fit between predictor and criterion as an explanation for the low predictive validity of implicit measures with regard to behavioral outcomes. More precisely, we argue that the predictive validity of implicit measures suffers from the fact that (1) studies often do not assess behavior proper but rather employ self-report measures as a criterion, and (2) implicit measures typically do not provide contextual information; details that are crucial for real-life behavior.

### Behavioral Intentions Versus Behavior Proper

Although the obvious criterion variable for a study on the predictive validity of implicit measures is behavior (e.g., actual discrimination), the assessment of behavior proper is by no means the rule. As has been prominently argued by Baumeister et al. (2007), measurement of actual behavior (a dominant approach during the 70s) in the field of social psychology has largely been superseded by “pseudo”-behavioral measures such as rating scale measures assessing behavioral intentions or past behavior. It is thus not surprising, that the same applies to studies assessing the predictive validity of implicit measures: Behavioral criteria in IAT studies often consist of self-report measures or similarly indirect indicators (e.g., Oswald et al., 2013; Carlsson and Agerström, 2016). Unfortunately, opting for self-report measures of behavior entails a number of shortcomings that are especially troublesome for testing the relationship of *implicit* measures and behavioral outcomes.

First, it has long been known that self-reported behavioral intentions are not an adequate proxy for actual behavior. For example, West and Brown (1975; for a detailed elaboration, see Baumeister et al., 2007) demonstrated a striking difference between participants’ intention to donate money for someone in need (participants were more than willing to help) and actual helping behavior (donations were close to zero). Second, indirect measures were conceived to overcome self-presentational concerns that typically affect self-report measures and/or to measure introspectively less accessible traces of experience. Consequently, relying on these very self-reports as the major criterion for predictive validity may have contributed to the heterogeneous landscape of findings on the validity of implicit measures.

What is more, we should probably refrain from referring to *behavior* as if it were a unitary construct. Instead, researchers should put forward specific hypotheses concerning the relationship of implicit measures, different types of behavior, and specific situational conditions. Dual-process or dual-systems models (e.g., Metcalfe and Mischel, 1999; Smith and DeCoster, 2000; Strack and Deutsch, 2004; Friese et al., 2008; Hofmann et al., 2009; Kahneman, 2011) provide a fine-grained view on this question and have frequently formed the basis for differentiation. These models essentially assume that there are different kinds of processes competing for behavior control. The processes differ with respect to the form in which information is stored and accessed, as well as the degree of conscious awareness and cognitive control involved. Though details and labels vary (e.g., automatic vs. controlled: Friese et al., 2008; hot vs. cool: Metcalfe and Mischel, 1999; associative vs. rule-based: Smith and DeCoster, 2000; impulsive vs. reflective: Strack and Deutsch, 2004), the common idea in these models is the distinction between two cognitive players. On the one hand, there is a system in which information is usually assumed to be stored and accessed in an associative manner. This system should operate fast, effortlessly and with little or no awareness and control. On the other hand, there is a second system in which information is assumed to be stored and accessed propositionally and which should drive controlled, slow and effortful deliberation. Both systems are hypothesized to compete for behavioral control, in a tug-of-war fashion, with motivation and opportunity for control as crucial moderators (e.g., Fazio and Towles-Schwen, 1999; Friese et al., 2008; Hofmann et al., 2009). While the first system is assumed to prompt spontaneous and impulsive behavior, the second should allow for reasoned action - but only if people are both motivated and able to spare the necessary cognitive resources (e.g., Hofmann et al., 2007; Friese et al., 2008). As a case in point, Pearson et al. (2009) summarize:

“Whereas explicit attitudes typically shape deliberative, well-considered responses for which people have the motivation and opportunity to weigh the costs and benefits of various courses of action, implicit attitudes typically influence responses that are more difficult to monitor or control […] or responses that people do not view as diagnostic of their attitude and thus do not try to control.” (p. 322).

A comprehensive overview of the more nuanced theoretical views on conditions under which implicit vs. explicit measures predict behavior is beyond the scope of this paper. For an overview of different models, we refer readers to Perugini et al. (2010). As for now, however, it is important to note that dual-systems models are not without criticism (e.g., Rothermund, 2011; Gawronski and Creighton, 2013). Some of their assumptions have even set confining boundaries and require revision. Especially the frequently deduced notion that implicit measures like the IAT would reflect associations and therefore predict impulsive behavior while explicit measures like self-reports would reflect propositional reasoning and therefore explain deliberate acts (e.g., Friese et al., 2008) is probably an oversimplification. As we noted in the section on implicit beliefs, some features of automaticity that had previously been reserved exclusively for associative processes also apply to propositional information. At the same time, ostensibly implicit measures like the IAT do not necessarily reflect purely automatic processes, as also outlined before. Instead, it might prove more useful to distinguish between the different processes that might be involved. In other words, to the extent that implicit measures tap into processes operating outside of cognitive control, they should relate to impulsive behavior. Thus, although some assumptions of these models might have been too strict, dual-process or dual-systems models have enriched the literature with inspiring hypotheses and findings. They have proven successful in integrating and organizing a large part of the literature on implicit and explicit measures and their relation to behavior. Indisputably, an important strength of these models lies in their differentiation between various forms of behavior. It is reasonable to assume different predictive power depending on the degree of cognitive control involved. So, when it comes to improving the predictive power of implicit measures, our call for differentiation also applies to the criterion variable: not all forms of behavior should be treated equal, and cognitive resources should be taken into account. Researchers are well advised not to simply explore whether an implicit measure predicts behavior, or whether it outperforms explicit measures in doing so. They should rather specify more sophisticated hypotheses on the kind of behavior that should be predicted (e.g., spontaneous behavior), or under which conditions (e.g., depleted self-control resources) such a relationship is to be expected.

To sum up, we want to highlight the notion that a robust estimation of implicit measures’ predictive validity critically hinges on the quality of the criterion. We therefore recommend to drop self-report measures and other indirect criterion variables in favor of actual, rather spontaneous forms of behavior.

### Context Dependency of Attitudes and Beliefs

Finally, it is important to realize that behavior is enacted in a specific situation or context (e.g., we react to someone *at work* vs. *in the family*). Therefore, behavior is inherently *context-specific*. In contrast, implicit measures in general do not specify contextual information and assess attitudes, stereotypes, or beliefs in a *context-independent*, global fashion. Aiming for such an assessment of “the” attitude (e.g., toward Blacks, women, gays, or the elderly) is also at odds with the finding that more or less all attitudes, beliefs, and stereotypes are context-specific (Blair, 2002; Wigboldus et al., 2003; Casper et al., 2010, 2011; Kornadt and Rothermund, 2011, 2015; Müller and Rothermund, 2012; Gawronski and Cesario, 2013). Consequently, assessing attitudes or beliefs in situational vacuum will often not be specific enough to predict a particular behavior toward a specific attitude object in a specific situation (Blanton and Jaccard, 2015).

### A Solution: Introducing the Context Into Implicit Measures

One possibility to address this gap in “level of detail” is to aggregate behavioral outcomes across different situations, time points, and target objects yielding a *context-independent* behavioral indicator in line with the context independent nature of implicit measures (e.g., of discriminatory behavior; Ajzen, 1991). Another and more economic possibility would be to increase the “structural fit” (Payne et al., 2008) between implicit measures of attitudes and the to-be-predicted situation-specific behaviors by introducing *context-specificity* also on the level of implicit measures of attitudes. This allows us to capture the heterogeneity of evaluations that an individual can harbor with regard to the same object (Gawronski et al., 2018), and it increases the chances to predict matching context-specific behaviors (e.g., Blanton and Jaccard, 2015). In this regard, measures employing dual primes incorporating both category and context information (Casper et al., 2010, 2011) or specifying context-dependent evaluative meanings when choosing attribute categories in the IAT (Kornadt et al., 2016) represent promising approaches for future research. Implicit measures of propositional beliefs (see Issue 3 above) are also well-suited in this regard since they allow researchers to clearly specify contextualized meanings in the stimulus materials. Similarly, the strength of the motivational drive to pursue specific incentives typically depends on context cues signaling their (un-)availability. For instance, individual differences in the strengths of motivational approach (or avoidance) tendencies regarding relationship initiation will be triggered in a dating context (Nikitin et al., 2019) but probably will not influence behavior toward men and women in the work context. Incorporating this context-specificity into implicit measures of wanting (see Issue 2 above) will thus be an important step to capture the determinants of our desires and to better explain and predict social behavior.

To summarize, assessing the potential of implicit measures for explaining and closing the attitude-behavior gap requires both predictors (implicit attitudes and beliefs) and criterion variables (e.g., discriminatory behaviors) to be assessed in a reliable, valid, and contextualized way. This necessitates both changes in implicit measures (to address the context-specificity of the to-be-measured constructs) as well as rigorous theorizing about which aspects of which type of behavior are to be influenced by (context-specific) attitudes and beliefs.

## Closing Thoughts

In this article, we presented an overview of possible reasons for the weak relationship between implicit measures like the IAT and behavioral criteria. We outlined that the unsatisfying predictive value of the IAT is due to (1) extraneous influences like recoding, (2) the measurement of liking instead of wanting, (3) the measurement of associations instead of complex beliefs, and/or (4) a conceptual mismatch of predictor and criterion. We presented precise solutions for each of these problems. More precisely, we suggested to switch to procedural variations that minimize extraneous influences (i.e., the SB-IAT, Teige-Mocigemba et al., 2008; and the IAT-RF; Rothermund et al., 2009), and to apply sophisticated analysis tools (i.e., the ReAL model, Meissner and Rothermund, 2013) that separate relevant processes from those extraneous influences. Second, we presented an overview of different implicit measures that go beyond the measurement of evaluative associations, and instead quantify actual implicit wanting (e.g., the W-IAT, Koranyi et al., 2017). Third, we pointed to implicit measures of beliefs (e.g., the PEP, Müller and Rothermund, 2019) that allow a more nuanced view on individual attitudes and values than measures that tap into associations. Finally, we emphasized the importance of measuring behavior proper and outlined that implicit measures incorporating contextual information might be more adequate in assessing the structure of implicit attitudes or beliefs and their implications for behavior (Casper et al., 2011; Kornadt et al., 2016). Each of the recent developments presented in the current paper has the potential to increase the predictive power of implicit measures. Future research will also have to clarify whether a combination of these approaches may lead to further improvement. Inspired by the fruitful research on dual-process or dual-systems models, we further suggest to invest in theoretical considerations: Which forms or aspects of behavior should be related to which processes involved in which implicit measures? Differentiation is key, with regard to both the predictor and the criterion.

We strongly argue not to take the validity of implicit measures like the IAT for granted. Instead, we should take into account the complexity of these measures, especially when it comes to the predictive value for real-life behavior. As outlined in the current review, the past 20 years of research have provided us with a number of good reasons for why the IAT and its derivatives did not succeed in closing the attitude-behavior gap, and enriched our toolbox with promising, sophisticated improvements. Future research will benefit from harnessing the power of such a more differentiated view on implicit measures.

## Author Contributions

FMe and KR wrote the first draft of the manuscript. LG, NK, and FMü wrote sections of the manuscript. All authors contributed to manuscript revision, read and approved the submitted version.

### Conflict of Interest

The authors declare that the research was conducted in the absence of any commercial or financial relationships that could be construed as a potential conflict of interest.

## Footnotes

^1^We are aware that a couple of researchers actually exercise due caution when interpreting IAT scores, understanding them as response time differences in a computerized categorization task – no more, no less. However, the majority of researchers do interpret IAT scores as reflecting associative strength or implicit bias. After all, the IATs very name suggests such an interpretation. In this paper, we therefore proceed from this more common viewpoint.

^2^The recoding account subsumes two earlier process models for the IAT: the so-called figure-ground account (Rothermund and Wentura, 2001, 2004; Rothermund et al., 2005; see also Chang and Mitchell, 2009; Kinoshita & Peak-O’Leary, 2006; Mitchell, 2004) and the task-switching account (Mierke and Klauer, 2001,^2003^; Klauer and Mierke, 2005). For an overview of these and other process accounts for the IAT we refer to the work of Teige-Mocigemba and colleagues (Teige-Mocigemba et al., 2010; Teige-Mocigemba and Klauer, 2015).

^3^Note that there is another procedure that dropped the IAT’s block structure, namely, the Extrinsic Affective Simon Task (EAST, De Houwer, 2003b; see also its close cousin, the Identification EAST, De Houwer and De Bruycker, 2007). Importantly, however, the EAST does not contain classification responses based on the target categories and is thus strongly susceptible to stimulus effects (Gast and Rothermund, 2010). Furthermore, it suffers from low reliability (De Houwer, 2003b). We therefore consider the EAST a less recommendable approach to account for the problem of recoding.

^4^Note that for many IATs, we do not know *a priori* which of the two blocks will be simplified by recoding. Even within one sample, some participants might recode the task in one IAT block (e.g., in the Black/positive block), others will do so in the other block (i.e., the White/positive block). The ReAL model accounts for these differences by making use of the task switch cost effect as a marker for recoding processes. More precisely, the sign of the individual switch cost effect determines the block in which the *Re* parameter is modeled (for more details, see Meissner and Rothermund, 2013, 2015b).
